# Direct Electrochemical Detection of Bisphenol A Using a Highly Conductive Graphite Nanoparticle Film Electrode

**DOI:** 10.3390/s17040836

**Published:** 2017-04-11

**Authors:** Xinwei Dong, Xiaoli Qi, Na Liu, Yuesuo Yang, Yunxian Piao

**Affiliations:** 1 Key Laboratory of Ground Water Resources and Environment of the Ministry of Education, College of Environment and Resources, Jilin University, 2519 Jiefang Road, Changchun 130021, China; dongxw14@mails.jlu.edu.cn (X.D.); qixl15@mails.jlu.edu.cn (X.Q.); liuna@jlu.edu.cn (N.L.); YangYuesuo@jlu.edu.cn (Y.Y.); 2 Key Laboratory of Eco-restoration of Regional Contaminated Environment, Shenyang University, Shenyang 110044, China

**Keywords:** Bisphenol A, adsorption, graphite nanoparticle, electrochemical oxidation

## Abstract

We developed an accurate and sensitive sensor for electrochemical detection of bisphenol A (BPA) with a high-conductivity graphite nanoparticle (GN) film electrode. The GNs consisted of several stacked graphene sheets and showed a homogenous spherical shape, high conductivity, large surface area and good adsorption properties to BPA. The constructed GN film electrode exhibited improved amperometric current responses such as decreased impedance and lowered BPA oxidation potential compared with those of a pristine electrode, and also possessed a large surface area to allow fast electron transfer and BPA accumulation. A pre-accumulation process with BPA adsorption resulted in considerable current signal enhancement during BPA detection. The loading amount of GNs on the film electrode and the time for target BPA enrichment were optimized. The GN film electrode-based sensor showed high reproducibility and high selectivity for BPA over other reagents. Differential pulse voltammetry experiments revealed that the concentrations of BPA were linearly correlated with the current changes, and the lowest limit of detection of the sensor was 35 nM. Furthermore, the sensor showed great accuracy and reliability, as confirmed by high-performance liquid chromatography measurements. The sensor was also successfully used for BPA determination in groundwater samples, demonstrating its potential for real environmental analysis.

## 1. Introduction

Bisphenol A (BPA) was used extensively in the manufacturing of polycarbonate plastics and epoxy resins [1,2,3,4]. However, BPA is an endocrine disrupting compound, has estrogenic activity, and may have adverse effects on humans, wildlife, and reproductive systems [5,6,7,8]. BPA is released into the environment through sewage treatment effluent, landfill leachate, and natural degradation of polycarbonate plastics [1,8]. Because of concern over the health risks of exposure to BPA, it is necessary to monitor trace amounts of BPA in the environment.

Traditional analytical methods such as liquid chromatography and gas chromatography [9,10] can achieve highly sensitive and precise detection of BPA. However, these methods can only be carried out in specific laboratories, and require highly trained technicians, time-consuming sample pretreatment processes, and expensive instruments. Thus, these methods are unsuitable for rapid and on-site detection. Recently, other advanced analytical methods like fluorimetry, enzyme-linked immunosorbent assay, and molecular imprinting have also been adapted for BPA determination [11,12,13]. Electrochemical methods also show great potential for in situ environmental monitoring because of inherent advantages such as fast response, low cost, ease of miniaturization, and high sensitivity and selectivity [14,15,16,17].

Various efforts have been made to improve the performance of direct electrochemical analysis for the detection of BPA. In particular, electrode construction with carbon nanotubes, fullerene (C_60_), graphene, and other carbon materials to increase surface area and conductivity has been attempted [18,19,20]. Most of these attempts focused on increasing electrode conductivity, which can not only improve detection sensitivity, but also lower the oxidation potential required for target molecule detection [1,21,22,23]. However, the sensing signal amplification by adsorption accumulation of target molecules on the conductive electrode has rarely been mentioned [24]. Furthermore, to obtain a robust and stabilized sensor, improving the physical and chemical stability of the electrode is one of the concerns, and the nano-sized carbon particle would be a good choice for electrode construction.

In this sense, we considered electrode construction with spherical graphite nanoparticles (GN), since it has favorable properties for direct electrochemical BPA analysis. The GNs consisted of about ten graphene sheets stacked in a layer-by-layer manner and the particle diameters were around 5 nm, which is comparable to the biomolecular size that we have previously studied [18]. These GNs would not only exhibit high conductivity because of their graphene-like features, and but also possess a large surface area to facilitate target molecule adsorption-accumulation and fast electron transfer. Furthermore, the nanoscale size of the GNs should be good for maintaining the properties and stability of GN film electrodes.

**Scheme 1:**
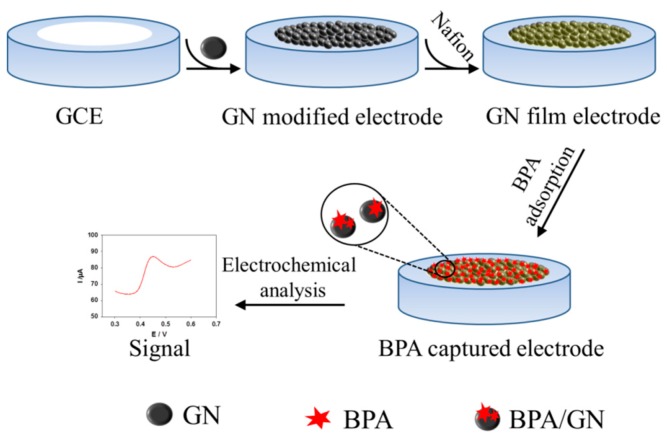
Representations of the procedures for graphite nanoparticle (GN) film electrode construction and the electrochemical detection of BPA.

In this study, our objective is to exploit the excellent properties of GNs to realize an advanced electrochemical sensing method for simple, highly sensitive, and reproducible BPA detection at low cost (Scheme 1). We explore the suitability of a GN film electrode as a direct electrochemical BPA sensor following adsorption-accumulation of BPA. The GN film electrode is also used for direct and rapid detection of BPA in groundwater samples.

## 2. Materials and Methods

### 2.1. Chemicals and Apparatus

Bisphenol A (BPA), K_3_Fe(CN)_6_ and a nafion solution (~5% in a mixture of lower aliphatic alcohols and water) were purchased from Sigma Aldrich (St. Louis, MO, USA). Graphite nanoparticles (93%, 540–650 m^2^·g^−1^ of specific surface area, 1.2–2.8 g·m^−3^ of true density, and 3–4 nm of average particle size) were obtained from Sky Spring Nanomaterials, Inc. (Houston, TX, USA). The stock solution of BPA was prepared in an anhydrous ethanol and was serially diluted with phosphate buffer (50 mM, pH 7.0) to specific concentrations before electrochemical analysis. All other chemicals were of analytical reagent grade and used without further purification.

The transmission electron microscope image (TEM) of GN was obtained using a high resolution transmission electron microscope (JEM-2100F, JEOL, Tokyo, Japan), and the scanning electron microscope image (SEM) of the GN film electrode was obtained using a XL-30 field emission scanning electron microscope (FEI, Hillsboro, TX, USA).

### 2.2. Preparation of GN Film Electrode

For preparation of the GN film electrode, firstly, a glass carbon electrode (GCE) with a 3-mm diameter (Shanghai Chenhua Instrument Co. Ltd., Shanghai, China) was polished with 0.5 μm alumina slurry. After brief washing with double distilled water and sonication for 3 min, it was followed by sequential washing with the water and ethanol, and drying under a nitrogen gas. Then, 7.0 μL of GN solution (3.5 mg·mL^−1^) prepared in double distilled water after sonication was deposited on the polished GCE surface, and incubated for 40 min under an ambient condition. After that, 2 μL of nafion (0.25%) was added onto the GN to produce a GN film on the GCE. The constructed GN film on the GCE was then ready to use as a working electrode in BPA sensing.

### 2.3. Electrochemical Measurements

Electrochemical performance of GN film electrode was determined using a CHI660E electrochemical workstation (Shanghai Chenhua Instrument Co. Ltd., Shanghai, China). Three electrode systems consisting of a GN film electrode, Ag/AgCl electrode, and Pt wire as working, reference, and counting electrodes, respectively, were used. Before the electrochemical analysis, 10 mL of BPA solution was purged with a purified nitrogen gas for 10 min. This was followed by incubating the GN film electrode in the BPA solution for 20 min for accumulation and enrichment. Then, the electrochemical analysis was performed with cyclic voltammetry or different pulse voltammetry. The electrochemical impedance spectroscopy (EIS) measurements were carried out in a 1 mM K_3_Fe(CN)_6_ solution containing 0.1 M KCl. All analytical measurements were performed at room temperature.

### 2.4. Real Environmental Water Sample Detection

Ground water sample was obtained from the vicinity of the South Lake, Changchun, China. Without any further treatment, the water sample was spiked with BPA, and the amount of BPA was quantified by different pulse voltammetry using the GN film electrode.

The BPA concentration was also quantified using the conventional HPLC method with an LC-20AT HPLC system (Shimadzu, Kyoto, Japan), that was equipped with a UV-vis detector at 278 nm and an Eclipse Plus C18 column (250 × 4.6 mm, particle size of 5 μm, Agilent, Santaclara, CA, USA). The mobile phase was composed of acetonitrile and deionized water with a volume radio of 50:50. The flow rate was set to 1.0 mL·min^−1^.

## 3. Results and Discussion

### 3.1. Physical and Electrochemical Properties of the GN Film Electrode

The morphology of the GN was evaluated by TEM analysis. As shown in Figure S1, the GN has spherical morphology and the average particle size was 5 ± 0.4 nm (*n* = 20). The surface property of the constructed GN film electrode was accessed by SEM analysis. As presented in Figure S2A, a homogeneous GN film on GCE was produced, which was attributed to the properties of small particle size and the good dispersity of the GN.

The electrochemical surface area of the GN film electrode was evaluated by performing the chronocoulometry in a 1 mM K_3_Fe(CN)_6_ solution, and calculated based on the following equation, as previously reported.

**Figure 1:**
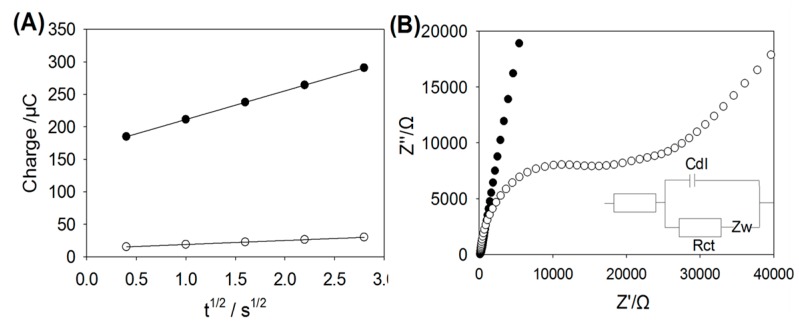
(**A**) Plot of *Q*-*t*^1/2^ curves on the GN film electrode (black circle) and the glassy carbon electrode (GCE) (white circle) in BPA (10 μM) solution; (**B**) Nyquist plots obtained at the GN film electrode (black circle), and the GCE (white circle) in 1 mM K_3_Fe(CN)_6_ and 0.1 M KCl with the frequencies swept from 10^5^ to 0.01 Hz. Inset: rundles equivalent circuit of the GCE and GN film electrode to fit the impedance data.

*Q* = 2*nFACD*^1/2^*t*^1/2^*π*^−1/2^

where *n* is the electron transfer number (*n* = 1 for K_3_Fe(CN)_6_), *A* is the effective surface area of the working electrode, *C* is the concentration of substrate, and *D* is the diffusion coefficient of K_3_Fe(CN)_6_ (*D* = 7.6 × 10^−6^ cm^2^·s^−1^). According to the slope values for the plot of Q vs. *t*^1/2^ in Figure 1A, the effective surface areas of the GCE and GN film electrodes were calculated to be 0.024 cm^2^ and 0.146 cm^2^, respectively, indicating that the effective surface area of the electrode increased greatly after modification with GN, which positively contributed to the enhancement of the current response at the detection of BPA.

Electron transfer properties of the GN film electrode were accessed by the EIS measurements. The electron transfer resistance (R_ct_) can be reflected by the semicircle diameters of the Nyquist plot. As shown in Figure 1B, the R_ct_ values at the GN film electrode obtained in 1.0 mM K_3_Fe(CN)_6_ solution was 0.07 Ω, which was much less than 495 Ω, at the GCE, implying a much lower charge transfer resistance of the GN film electrode, that is due to the incorporation of the high conductivity GN.

### 3.2. Electrochemical Response to BPA

**Figure 2:**
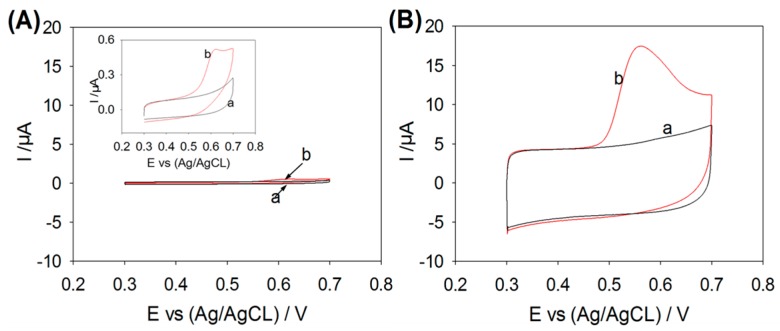
Cyclic voltammetric responses of (**A**) the GCE and (**B**) the GN film electrode in (a) absence and (b) presence of BPA (10 μM) solutions.

The electrochemical behavior of the GN film electrode response to BPA was investigated by the cyclic votalmmetric analysis. As shown in Figure 2, without BPA addition, there was no current peak observed with both the GCE and GN film electrode. When there was BPA (10 μM), a BPA oxidation peak current at a potential of 0.621 V was presented with the GCE electrode, where the current shift was 0.27 μA (Figure 2A). In contrast, with the GN film electrode, the BPA oxidation peak current was observed at a lower potential of 0.563 V with the current shift of 12.28 μA (Figure 2B). The lowered anodic potential and the significantly amplified current value (45-fold higher than with the GCE) with the GN film electrode compared to the pristine GCE were due to the high conductivity performance and the faster electron transfer rate of the GN. This suggested that the GN could be used for high performance electrochemical sensing.

### 3.3. Scan Rate Effect

The influence of the scan rate on the electrochemical behavior of 10 μM BPA at the GN film electrode was also investigated (Figure S3A). It was observed that the cyclic voltammetric peak currents increased linearly with the scan rates (*v*) in the range of 10–90 mV·s^−1^ (Figure S3B), where the linear regression equation was *I*_pa_ (μA) = 0.27*v* (mV·s^−1^) + 1.379 (*R*^2^ = 0.993). This suggested that the BPA oxidation is an adsorption-controlled electrode process.

The peak potential (*E*_pa_) positively shifted with the scan rate increase (Figure S3C). The *E*_pa_ and the Napierian logarithm of the scan rate was linearly correlated with the regression equation of *E*_pa_ (V) = 0.026ln*v* + 0.472 (mV·s^−1^, *R*^2^ = 0.992). For a typical adsorption-controlled irreversible electrode process, *E*_pa_ was defined by the following equation:

*E*_pa_ = *E*^0^ + (*RT*/*αnF*)ln(*RTK*^0^/*αnF*) + *RT*/*αnF*ln*v*

where *α* is the transfer coefficient, *R* is the gas constant (*R* = 8.314 J·mol^−1^·k^−1^), *T* is the temperature (*T* = 298 K), and *F* is the Faraday constant (*F* = 96480 C·mol^−1^), respectively. According to the above equation, the slope of the line was equal to *RT*/*αnF*, so *αn* was calculated to be 0.99. For irreversible process, *α* was considered as 0.5, so *n* = 2, indicating that the electron transfer number was around 2.

### 3.4. Adsorption Effect of the GN Film Electrode

**Figure 3:**
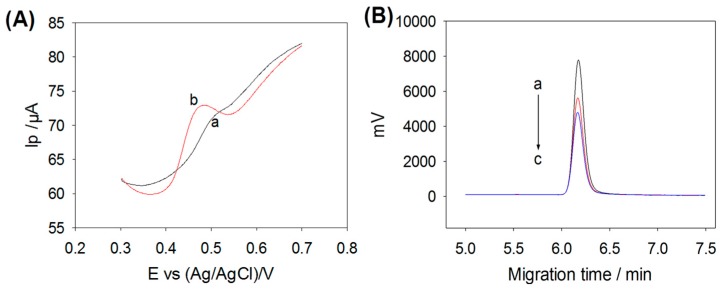
(**A**) Different pulse voltammetry responses of the GN film electrode in BPA (10 μM) solution, (a) without and (b) with accumulating process; (**B**) HPLC chromatograms for the supernatant obtained by incubating various amounts of GNs in BPA (100 μM) solutions for 20 min. a–c represent 0, 100 and 200 μg of GN, respectively.

Figure 3 illustrates the effect of the BPA accumulation process on the performance of the BPA sensing with the differential pulse voltammetry. As shown in Figure 3A, the anodic current was highly improved with the 20 min accumulation process when compared to that with the zero minute accumulation. To further investigate the accumulation process, various concentrations of GN (0, 100 and 200 μg·mL^−1^) were incubated with BPA (10 μM) in phosphate buffer (PB) for 20 min and, after centrifugation and separation, the remaining BPA in the supernatant was analyzed with the HPLC method. As shown in Figure 3B, the characteristic peak of BPA decreased as the amount of added GNs elevated, suggested that BPA can be adsorbed to GN, and this may contributed to a highly enhanced signal during BPA detection.

To investigate whether the BPA sensing signal enhancement was mainly attributed to the GNs, a comparison of experiments with the nafion-modified GCE (GCE/nafion) and the pristine GCE were performed, respectively. As shown in Figure S4, the BPA sensing current with the GCE/nafion (0.25% nafion) was 0.94 μA, which was a little higher than that with the GCE (0.77 μA), possibly because of the enrichment of BPA by the nafion. However, the current signal with the GN film electrode (12.28 μA) was about 13-fold higher than that with the GCE/nafion. This suggested that the current signal increase mainly contributed to the unique characteristics of the GN film electrode, such as the high conductivity and the high adsorption of BPA. 

### 3.5. Optimization of Parameters

**Figure 4:**
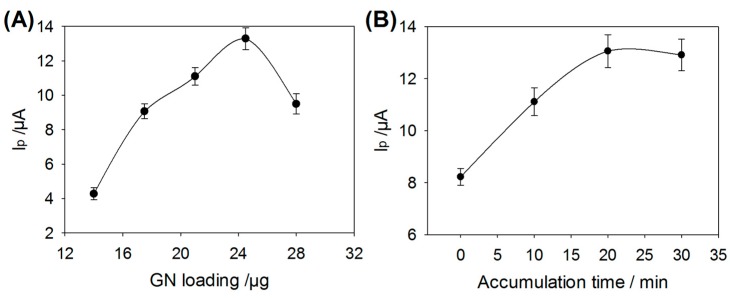
(**A**) The relationship between BPA oxidation peak currents and the amounts of GN loading (14–28 μg) on the GN film electrode; (**B**) The effect of pre-accumulation times on the BPA oxidation peak currents by the GN film electrode.

To obtain the GN film electrode with the most favorable electrochemical sensing property, the amount of loaded GN during the film electrode construction was optimized. As shown in Figure 4A, the BPA (10 μM) oxidation peak current gradually increased with the increase of the GN loading amount from 14 to 24.5 μg, which suggested that the increment of the GN loading can contribute to elevated current sensing signal. However, there was an oxidation current decrease as the amount of the GN loading elevated further to 28 μg. This is because a too thick layer of GN film would result in the adsorbed molecules being too far away to transfer electrons. In light of this, we chose to employ 24.5 μg of GN loading in the following experiments.

The accumulation time for target BPA detection was also optimized. As indicated in Figure 4B, a maximum current signal was obtained when the accumulation time was 20 min. And after 20 min the current signal was almost changeless, which suggested that the adsorption accumulation had reached saturation. For further studies, the accumulation time of 20 min was used.

### 3.6. BPA Detection Sensitivity

**Figure 5:**
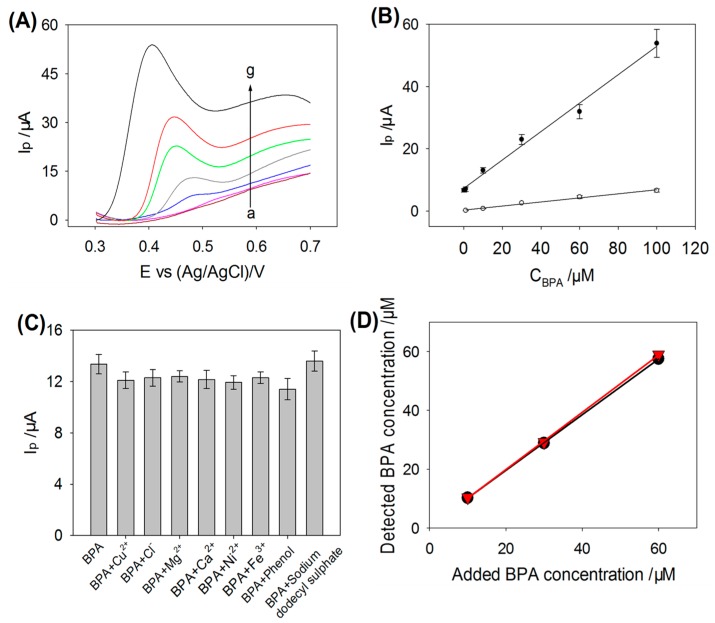
(**A**) Different pulse voltammetry profiles of the GN film electrode for detection of various concentrations of BPA in phosphate buffer (50 mM, pH 7.0) solution. a-g represent 0, 0.1, 1, 10, 30, 60 and 100 μM of BPA, respectively; (**B**) The calibration curves of the oxidation currents versus the BPA concentrations obtained using the GN film electrode (black circle) and the GCE (white circle); (**C**) Selectivity of the GN film electrode for detection of BPA (10 μM) amid the existence of irrelevant reagents; (**D**) Comparison of BPA detection results by the GN film electrode (black circle) with that by the conventional HPLC method (red triangle).

For the quantitative detection of BPA, the analytical method of differential pulse voltammetry was applied under the optimized conditions. As indicated in Figure 5A, the oxidation current increased as the concentrations of BPA elevated from 0.1 to 100 μM. The current values were linearly correlated with the BPA concentrations, with the linear regression equation of *ΔIp* (μA) = 0.4565*C*_BPA_ (μM) + 7.285 (*R*^2^ = 0.991) (Figure 5B). The sensitivity with the GN film electrode was 0.46 μA·μM^−1^·cm^−2^, which was seven-fold higher than that with the GCE, where the sensitivity was 0.07 μA·μM^−1^·cm^−2^. This suggested that the highly advantageous features of the GN film electrode with a large surface area, high adsorptive and conductivity properties would contribute to the greatly sensitive BPA detection. In addition, the lowest limit of detection with the GN film electrode was estimated to be 35 nM by the signal to noise ratio of three, which was lower than the value of predicted no effect concentrations for drinking water quality of China (GB 5749-2006) [25] and comparable with some others reported with the direct electrochemical analysis method [26,27,28,29,30,31,32] (Table S1).

### 3.7. Stability, Reproducibility and Selectivity of the GN Film Electrode

The stability of the GN film sensor was studied by storage of the GN film electrode at room temperature condition for months. It was found the BPA sensing signal was maintained at 95.7% of the initial signal after three months’ storage, suggesting that there was rarely a loss of the sensing signal, and that the GN film electrode has good stability even when stored at the ambient conditions. These findings contribute to the determination of physical and chemical stabilities of the GN film, including a rare loss of conductivity and adsorption capability. In addition, no film detachment was observed on the surface of the GCE through the whole experiment.

The reproducibility of the sensor was accessed by performing electrochemical analysis of BPA (10 μM) using five individual GN film electrodes. The relative standard deviation was calculated to be 7.1%, indicating that the proposed sensor has good reproducibility.

To investigate the selectivity of the GN film electrode, some potential interfering substances in PB solution containing BPA (10 μM) were taken into consideration. As shown in Figure 5C, 10-fold higher concentrations of some ions, such as Pb^2+^, Ni^2+^, Cu^2+^, Mg^2+^, Ca^2+^, Hg^2+^, Fe^3+^, SO_4_^2−^, Cl^−^, and HPO_4_^2−^, and five-fold concentrations of phenol and sodium dodecyl sulphate, indicated no obvious influences on the signals for BPA determination. Because of the good performances in reproducibility, stability and selectivity, the GN film sensor can be applied to real environmental water analysis.

### 3.8. Real Environmental Water Detection

In order to evaluate the applicability of the proposed sensor for real environmental water detection, the raw ground water samples were spiked with BPA to specific concentrations. The BPA concentrations in the treated samples were quantified according to the proposed sensing method under the optimized conditions. As shown in Table 1, for BPA concentrations of 10, 30 and 60 μM, recoveries of 102.7%, 96.13% and 95.83% were obtained with relative standard deviations of 2.23%, 2.43% and 2.82%, respectively, indicating that the GN film sensor is applicable in real environmental water analysis. In addition, the accuracy of the sensor was also evaluated by performing the HPLC method for detection of the same BPA levels in the ground water. As shown in Figure 5D, BPA concentrations measured by the GN film sensor were almost 100% overlapped by the results obtained by the HPLC method with recoveries of 104.3%, 97.0% and 98.2% for 10, 30 and 60 μM BPA, respectively. This suggested that the precision of the GN film sensor is reliable and has great potential as a sensitive, low cost, simple and fast alternative in field application.

## 4. Conclusions

With nano-sized and highly conductive graphite nanoparticles, a homogeneous and stable GN film electrode was produced. The GN film electrode showed higher conductivity property than the GCE, which resulted in a seven-fold increment in BPA detection sensitivity in contrast to the GCE. The graphite nanoparticle also had a large surface area for BPA adsorption, which contributed to the improved signal amplification for BPA analysis. The developed GN film sensor was investigated and found to not only have good reproducibility, stability and selectivity, but it also proved to have reliable accuracy as compared to the HPLC method. In this sense, the GN film sensor could be successfully applied for real environmental water analysis with good recoveries, implying its further broad applicability in the various environmental analyses.

## Figures and Tables

**Table 1:** Determination of BPA in real ground water samples by GN film electrode-based sensing method.

Added (μM)	Detected (μM) ^a^	RSD (%)	Recovery (%)
**10**	10.27	2.23%	102.7
**30**	28.84	2.43%	96.13
**60**	57.50	2.82%	95.83

^a^ Mean of three measurements.

## Supplementary Materials

Supplementary Materials can be found at http://www.mdpi.com/1424-8220/17/4/836/s1.
