# Supplementary material for: Direct Electrochemical Detection of Bisphenol A Using a Highly Conductive Graphite Nanoparticle Film Electrode
Source: Sensors (Basel). 2017 Apr 11;17(4):836. doi: 10.3390/s17040836 (PMC5422197; doi:10.3390/s17040836)
Supplement: Supplementary file 1 [file sensors-17-00836-s001.pdf]

Supplementary Materials: Direct Electrochemical Detection of Bisphenol A Using a Highly Conductive Graphite Nanoparticle Film Electrode

Xinwei Dong, Xiaoli Qi, Na Liu, Yuesuo Yang and Yunxian Piao

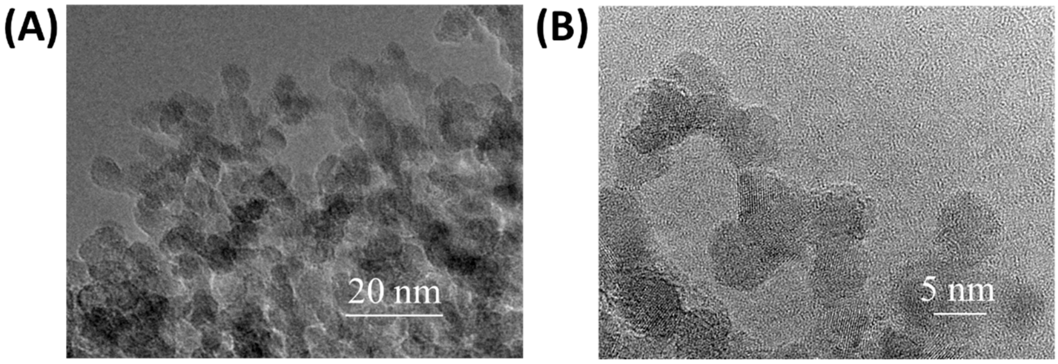

Figure S1. (**A**,**B**) TEM images of the GNs. The GNs are spherical carbon nanoparticles with a diameter of around 5 nm.

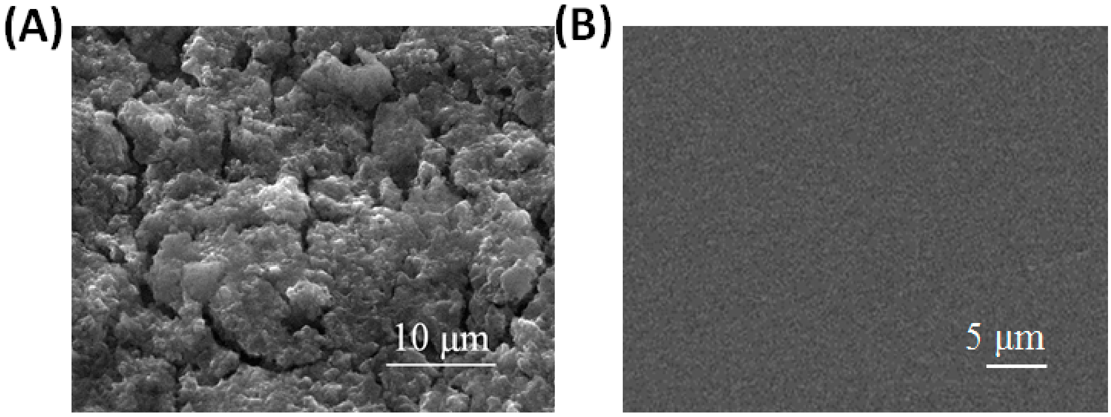

Figure S2. SEM images of (**A**) the GN film electrode and (**B**) the pristine GCE.

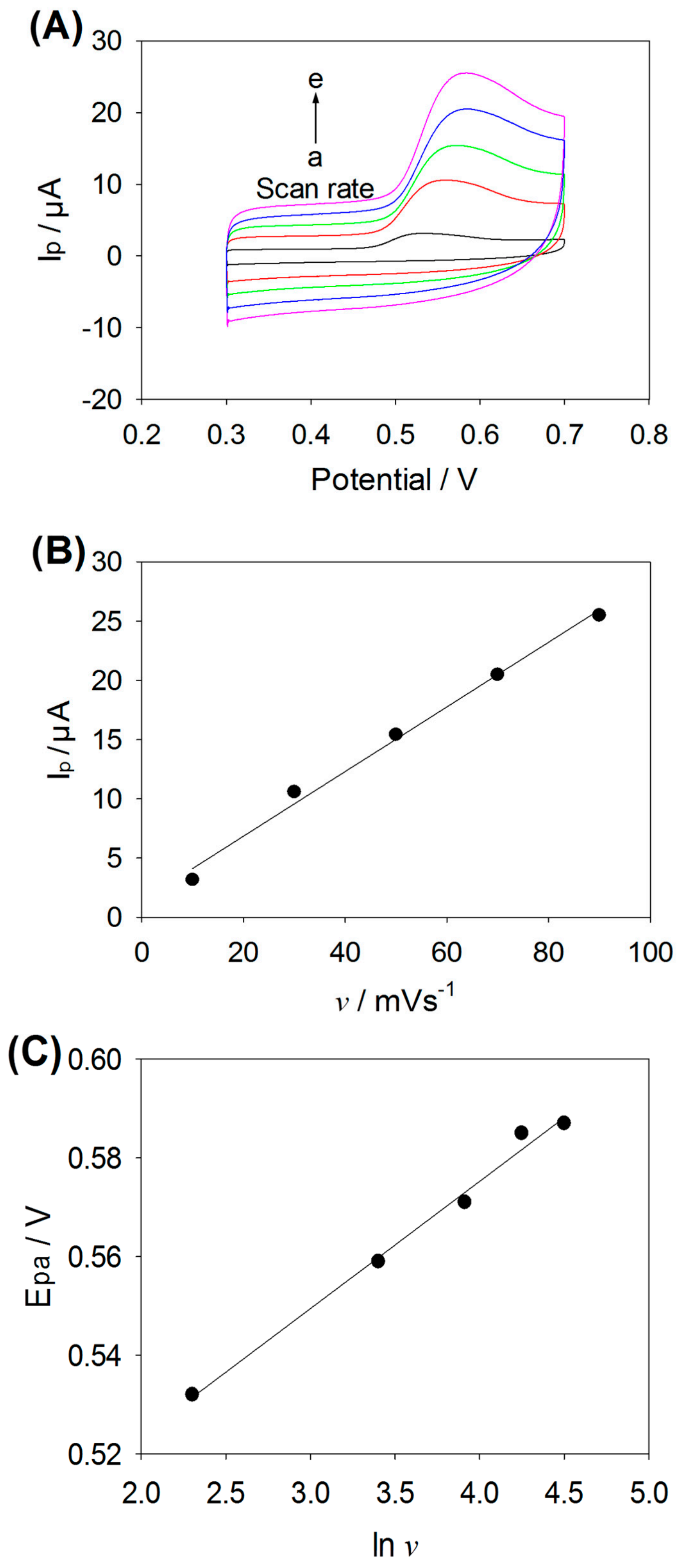

Figure S3. (**A**) Cyclic voltammetric responses of GN film electrode in BPA (10 μM) solution at varying scan rates (10, 30, 50, 70, and 90 $mV{\cdot}s^{-1}$). (**B**) The plot of peak current versus scan rate. (**C**) The relationship between the peak potential and the natural logarithm of scan rate. The arrow represents scanning direction.

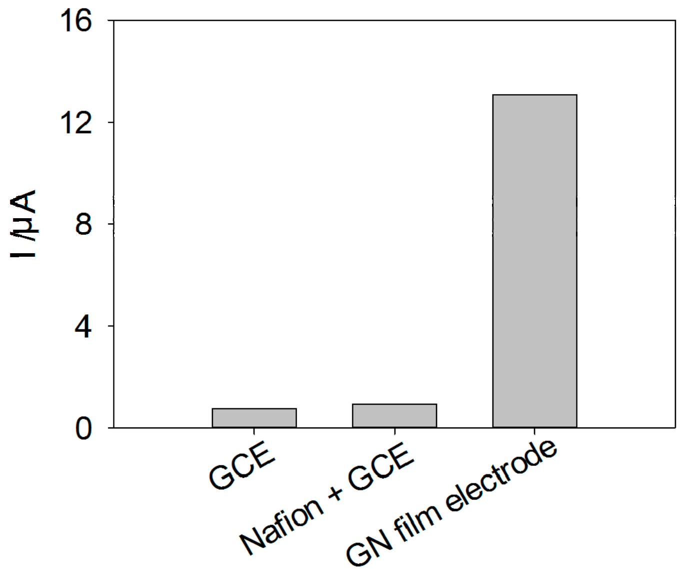

Figure S4. Electrochemical current responses of the GCE, the nafion-modified GCE electrode (GCE/nafion), and the GN film electrode in BPA solution (10 μM) with 20 min pre-accumulation process.

Table S1. Comparison with other reports in the literature of cases using the direct electrochemical analysis method.

Electrodes	Linear range (M)	Detection limit (M)	References
Fe_3O_4 NPs-Si4Pic+Cl−/Au NPs-Si4Pic+Cl−/GCE	0.2×10^{-7}–1.40×10^{-6}	7×10^{-9}	[26]
AuPdNPs/GNs	5.0×10^{-7}–1×10^{-5}	8×10^{-9}	[27]
r-CNTs/GCE	6.0×10^{-8}–8.0×10^{-5}	4.2×10^{-8}	[28]
Graphene/GCE	5.0×10^{-8}–1.0×10^{-6}	4.7×10^{-8}	[29]
AuNPs/SGNF/GCE	8.0×10^{-8}–2.5×10^{-4}	3.5×10^{-8}	[30]
MCM-41 sensor	2.2×10^{-7}–8.8×10^{-6}	3.8×10^{-8}	[31]
MIPPy/GQDs electrode	1.0×10^{-7}–5×10^{-5}	4×10^{-8}	[32]
MWCNTs-TiN/GCE	1.0×10^{-7}–5×10^{-5}	5×10^{-8}	[33]
GN film electrode	1.0×10^{-7}–1×10^{-4}	3.5×10^{-8}	Present work
